# Case Report: Adjuvant image-guided radiation therapy reduces surgical invasiveness in malignant peripheral nerve sheath tumors

**DOI:** 10.3389/fonc.2023.1129537

**Published:** 2023-04-21

**Authors:** Mandara M. Harikar, Gianluca Ferini, Paolo Palmisciano, Muhammad Shakir, Paolo Amico, Stefano Ferraresi, Giuseppe E. Umana

**Affiliations:** ^1^ Department of Neurosurgery, Trauma Center, Gamma Knife Center, Cannizzaro Hospital, Catania, Italy; ^2^ Department of Radiation Oncology, REM Radioterapia srl, Viagrande, Italy; ^3^ Department of Neurosurgery, University of Cincinnati College of Medicine, Cincinnati, OH, United States; ^4^ Section of Neurosurgery, Department of Surgery, Aga Khan University Hospital, Karachi, Pakistan; ^5^ Department of Pathological Anatomy, Cannizzaro Hospital, Catania, Italy; ^6^ Department of Neurosurgery, Ospedale Santa Maria della Misericordia, Rovigo, Italy

**Keywords:** malignant peripheral nerve sheet tumor, image-guided radiation therapy, radiotherapy, radiation oncology, sarcoma

## Abstract

**Introduction:**

Malignant peripheral nerve sheath tumors (MPNSTs) are a group of rare soft tissue sarcomas of mesenchymal origin. These tumors generally require extensive local excision owing to their aggressive potential. Though the role of radiotherapy is controversial, in this report, we present the case of an MPNST in the forearm that was treated with microsurgery followed by image-guided radiation therapy to achieve complete tumor disappearance at the 18-month follow-up.

**Case report:**

A 69-year-old woman with underlying paranoid schizophrenia was referred to our department with pain, severe swelling, and ecchymosis of her right forearm. Physical examination showed hypoesthesia in the segments innervated by the median nerve and reduced motor strength of her right hand. A gadolinium-enhanced MRI showed a large malignant peripheral nerve sheath tumor (13 x 8 x 7 cm) of the median nerve in the forearm. She underwent microsurgical en-bloc tumor resection with sparing of the median nerve. Thirty-five days postoperatively, she underwent image-guided radiotherapy (IGRT) using volumetric modulated arc therapy (VMAT). Serial MRI scans of the forearm with Gadolinium and whole-body CT scan with contrast enhancement at 30 days, 6 months, 1 year, and 18 months postoperatively documented no tumor recurrence, remnants, or metastases.

**Conclusions:**

In this report, we demonstrate the successful use of advanced radiotherapy techniques such as IGRT while avoiding demolitive surgery for MPNST. Though a longer follow-up is necessary, at the 18-month follow-up, the patient demonstrated good outcomes from surgical resection followed by adjuvant RT for MPNST in the forearm.

## 1 Introduction

Malignant peripheral nerve sheath tumors (MPNSTs) are a group of aggressive soft tissue sarcomas (STS) of mesenchymal origin. These tumors are characterized by high relapse rates, chemoresistance, and rapid progression, making them challenging to treat (1). Therefore, they generally warrant multidisciplinary intervention by surgeons, and medical and radiation oncologists (2).

Given the notoriously high rates of recurrence of MPNSTs and the difficulty in complete surgical extirpation due to tumor location, radiotherapy can be a useful treatment modality to achieve local tumor control (3, 4). However, the complications of RT, including fractures of adjacent bones and injury to critical neurovascular structures in the forearm, remain a serious concern (5).

The complex regional anatomy of the upper extremity poses additional challenges in the treatment of MPNSTs. First, it necessitates close surgical margins that are associated with the risk of residual disease and local recurrence (6). Second, it hinders the precise delivery of high radiation doses in the adjuvant or neoadjuvant radiotherapy setting (7). This can be circumvented by immobilization systems for accurate and reproducible positioning, rigorous set-up verification, and meticulous planning to ensure precise RT delivery (8). Furthermore, for improved planning, techniques such as volumetric modulated arc therapy (VMAT) that allow for accurate dose sculpting are now widely available.

Complete surgical resection forms the mainstay of treatment for MPNSTs. The role of radiotherapy in the treatment of MPNSTs, however, is widely debated. While several studies have shown that radiotherapy helps achieve margin control (9) and prolongs survival in soft tissue sarcomas (10–12), others have shown no impact on survival (13–15). There is also currently limited literature on the management MPNSTs in the literature, particularly that of the extremities.

In this report, we present a case of a 69-year-old woman with MPNST of the right forearm who underwent adjuvant RT after microsurgical resection, emphasizing the role of RT in minimizing the requirement for radical surgery.

## 2 Case report

**Figure 1:**
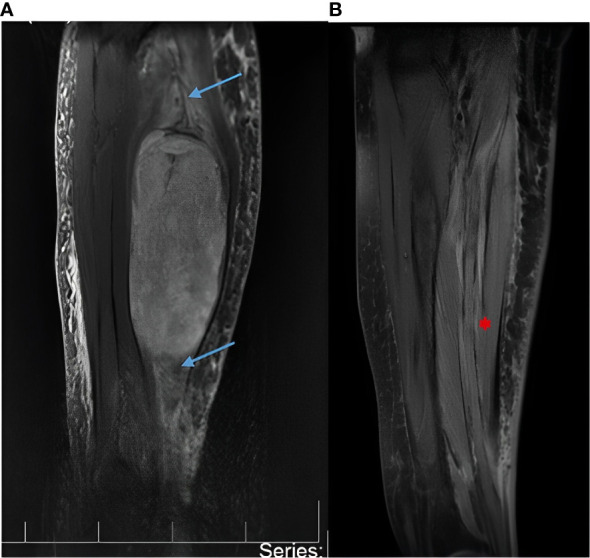
**(A)** Magnetic resonance imaging (MRI) with gadolinium showing a large malignant peripheral nerve sheath tumor (13x8x7cm) of the median nerve at the level of the forearm. The blue arrows show the median nerve proximal and distal to the tumor. The nerve appears enlarged due to the tumor growing in its context. The white arrow shows the tumor. **(B)** Postoperative MRI with gadolinium at 30 days postoperatively and every 6 months thereafter documented absence of tumor recurrence or remnants. The red arrow shows postoperative integrity of the median nerve.

A 69-year-old woman was referred to the Department of Neurosurgery at Cannizzaro Hospital, Catania, Sicily, complaining of pain and severe swelling associated with ecchymosis of her right forearm. The patient was on treatment for underlying paranoid schizophrenia. On physical examination, hypoesthesia in the segments innervated by the median nerve and reduced motor strength of her right hand (Medical Research Council, MRC grade 1/5) were noted. A gadolinium-enhanced MRI showed a large tumor (13 x 8 x 7 cm) arising from the median nerve at the level of the forearm (**Figure 1**).

**Figure 2:**
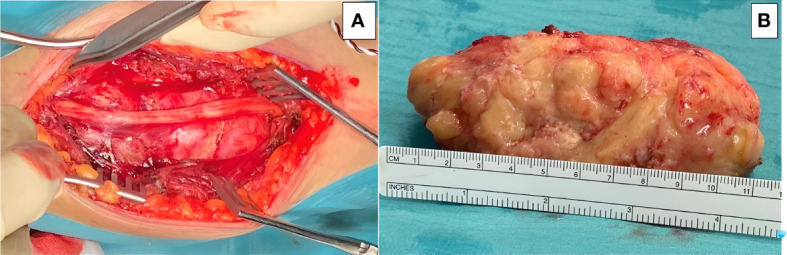
Microsurgical resection of the tumor was accomplished, and the lesion was removed enbloc: **(A)** The main trunk of the median nerve is seen running over the tumor surface. **(B)** Gross appearance of the tumor after resection, placed next to a ruler for scale.

The patient underwent microsurgical en-bloc tumor resection (**Figure 2**). The median nerve fibers were spared during surgery. Radical resection was achieved, but by microsurgical technique rather than compartmental approach so as to reduce surgical morbidity, thus sparing the function of the healthy surrounding tissue, including the parental nerve, surrounding muscles, tendons, and subcutaneous tissue.

**Figure 3:**
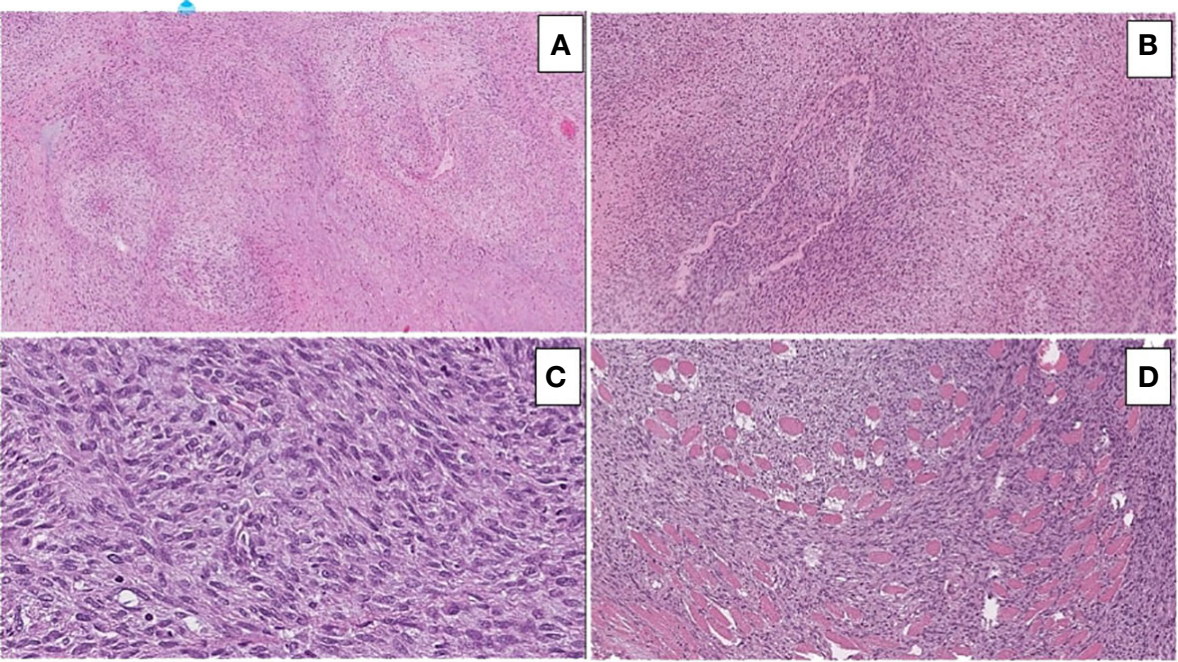
Hematoxylin and eosin-stained sections illustrating the main morphological findings: **(A)** On low power (10x magnification), classic marbled appearance due to alternating hypocellular and hypercellular areas with perivascular distribution of neoplastic cells is seen. **(B)** Hypercellular areas of the neoplasm consisting of uniform spindle cells with hyperchromatic, thin, and wavy nuclei are visible. **(C)** Nuclear atypia and numerous mitotic figures are easily identifiable. **(D)** Infiltration of the surrounding skeletal muscle is seen.

On histopathological examination, the tumor depicted a classic marbled appearance due to alternating hypocellular and hypercellular areas with perivascular distribution of neoplastic cells. The hypercellular areas of the neoplasm comprised uniform spindle cells with hyperchromatic, thin, wavy, nuclei. Nuclear atypia, numerous mitotic figures, and surrounding skeletal muscle infiltration were observed (**Figure 3**).

The immunohistochemical analysis of the tumor showed diffuse positivity for vimentin and INI1 and focal positivity for desmin, S100, GFAP, CD99, and CD34. It was negative for a-actin, HMB45, caldesmone, calponin, MYF4, CKAE1/AE3, and EMA. The morphological and immunophenotypic findings were compatible with the diagnosis of a malignant peripheral nerve sheath tumor (MPNST) stage III with focal rhabdomyoblast differentiation.

**Figure 4:**
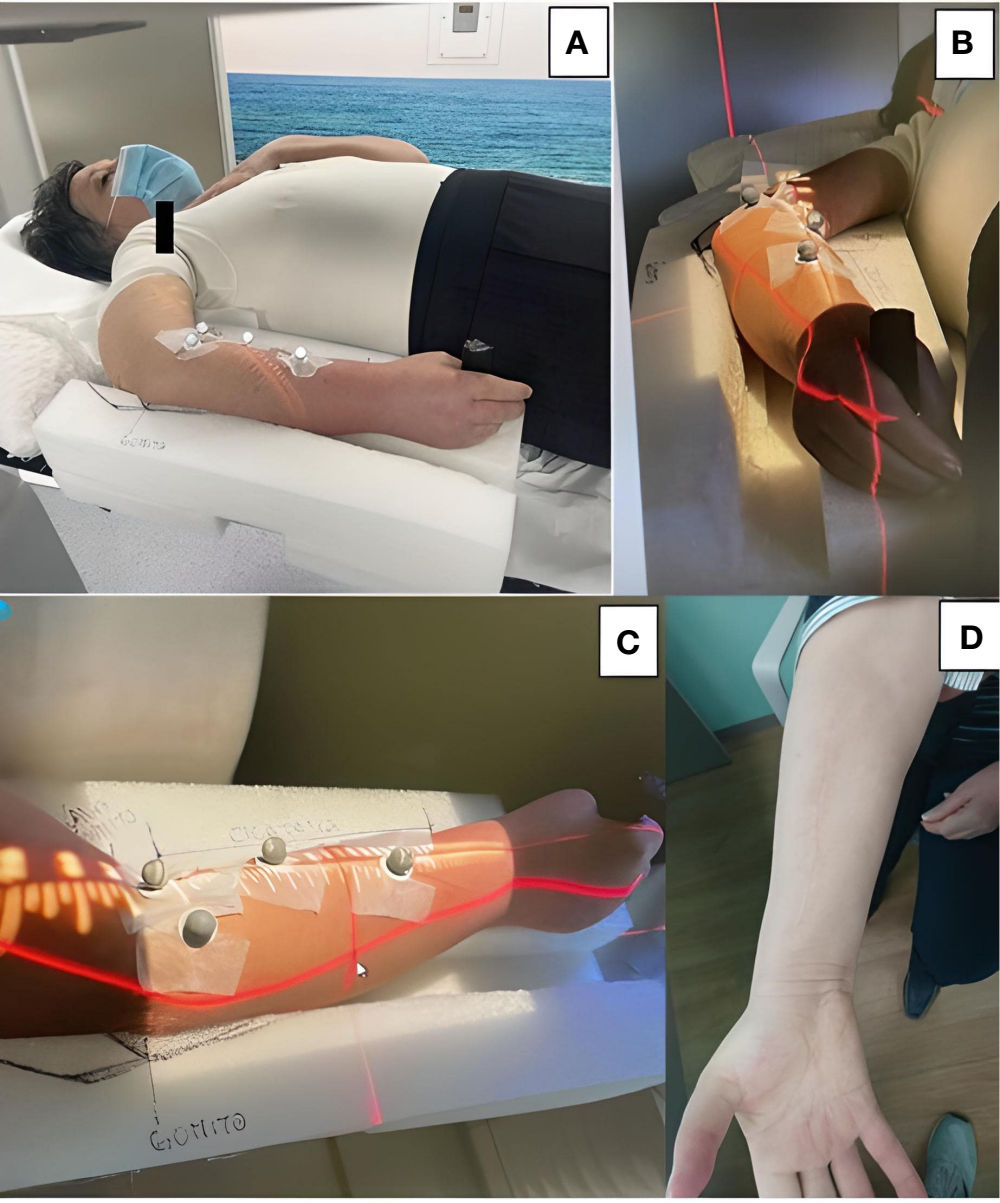
**(A-C)** A customized immobilization system was created by carving out foamed polyethylene. The patient gripped a cylindrical handle placed at the distal end for increased stability. **(B, C)** Four reflective body markers placed on the volar surface of the forearm were monitored by two ceiling-mounted infrared cameras for any intra-fraction target motion. **(D)** Satisfactory cosmetic result at the 1-year follow-up.

Thirty-five days postoperatively, the patient was posted for adjuvant RT following R1 resection of the tumor. The clinical target volume was decided as the whole tumor bed including the entire flexor muscle compartment of the right forearm, extending from the elbow to the wrist. The tumor bed was irradiated with a total dose of 70 Gy in fractions of 2 Gy/day under ExacTrac image guidance as described by Ferini et al. (8) (**Figure 4**). To avoid excessive radiation-induced injury to the radius and ulna, which closely abutted the high-dose radiotherapy target, we adopted an image-guided radiotherapy (IGRT)-volumetric modulated arc therapy (VMAT) approach rather than three-dimensional conformal radiotherapy verified through classic low-contrast resolution kilovoltage imaging systems or megavoltage electronic portal imaging devices.

The patient did not require chemotherapy. Serial MRI scans of the forearm with Gadolinium and whole-body CT scan with contrast enhancement at 30 days, 6 months, 1 year, and 18 months postoperatively documented no tumor recurrence, remnants, or metastases (**Figure 1**). She fully recovered her motor strength, with only mild hypoesthesia reported in the lateral three fingers of the right hand. At the latest follow-up in Sep 2022, no recurrence or toxicities were observed. The patient timeline is depicted in **Supplementary Figure 1**.

## 3 Discussion

In this report, we present the successful treatment of MPNST in the forearm by microsurgical resection followed by adjuvant radiotherapy, which led to excellent oncological, functional, and cosmetic outcomes.

MPNSTs may be sporadic or occur secondary to NF1 or prior radiation therapy (16, 17). Neurofibromatosis is the most important risk factor for MPNST, with 50–60% of the tumors occurring in those with NF-1 (13). However, our patient did not show features suggestive of NF-1 and had no history of radiotherapy, thus suggesting a sporadic occurrence. Though the prognostic significance of MPNST etiology remains undetermined, the large size of the tumor, deep location, and rhabdomyoblastic differentiation were among the poorer prognostic factors in our patient (18).

Though cases of MPNST in the forearm have been reported in the literature, they have mainly been in the context of unusual tumor presentation (19–22) surgical excision (23), or reconstruction (24, 25). To the best of our knowledge, this is the first clinical case report of MPNST in the forearm focusing on the potential of RT in minimizing the need for radical surgery. Extensive surgeries may result in adverse functional, cosmetic, and psychological consequences, and despite the disfiguring nature of the procedure, may still lead to poor outcomes (26). In this case report, we wish to highlight that adding novel radiotherapy techniques can improve the management of patients who previously had a chance of treatment only with compartmental surgery. In other words, though surgery with wide resection margins is the mainstay of treatment for MPNSTs, it is often unable to obtain complete remission; rather, it can only delay the final outcome, especially in large tumors as in this case.

Further, we employed microsurgical techniques to achieve radical resection without breaching the thin pseudo capsule of the tumor. This is of special interest especially in this patient with a psychiatric illness, who could not have accepted any demolitive surgery and already considered her sister guilty of the disease. A demolitive surgery, with its associated cosmetic alteration, would have been poorly tolerated by the patient. We were also able to achieve complete functional restoration of the upper limb, which is imperative for good quality of life.

Sarcomas have traditionally been considered to be radioresitant; however, RT has now been routinely incorporated into sarcoma treatment regimens for more than a century (27). The introduction of newer RT modalities, such as IGRT, intensity modulation RT, volumetric modulated arc therapy, stereotactic RT, and proton-based RT has revolutionized the field of radiation oncology, while resulting in fewer toxicities (8, 27, 28). Given the locally aggressive nature of the tumor and risk of local and distant recurrence, several authors recommend surgery with concomitant radiotherapy as the standard of care for MPNSTs (3, 4, 14, 29–32).

We employed the use of the ExacTrac imaging system, a customized immobilization device, and VMAT to reduce unintended radiation exposure to the forearm bones (8). Unlike classic treatment verification systems, ExacTrac allowed real-time indirect monitoring of the target setup during the daily radiation dose delivery through an infrared-based optical positioning system, using markers placed on the volar surface of the patient’s forearm. Such intra-treatment monitoring was fundamental considering the high mobility of this site. The trifecta of correct timing of the radiotherapy and delivery of high doses using new techniques that minimize the risks of side effects is a viable option that deserves further investigation.

One of the limitations of the report would be the relatively short period of follow-up (18 months). Though regular follow-up of the patient is still ongoing, prospective studies examining the safety and effectiveness of RT in larger cohorts are warranted. Considering that MPNST is a rare tumor and its treatment is still contentious, this report makes a useful contribution to the literature by underscoring the importance of RT in achieving remission after non-demolitive surgery.

In conclusion, MPNSTs in the forearm are challenging to treat; however, advances in radiotherapy techniques can be harnessed in conjunction with microsurgery to achieve complete tumor remission.

## 4 Patient’s perspective

The patient provided fully informed consent for the publication of this report and the accompanying images. The patient is highly satisfied with the oncological, functional, and cosmetic outcomes obtained.

## Data availability statement

The original contributions presented in the study are included in the article/**Supplementary Material**. Further inquiries can be directed to the corresponding author.

## Ethics statement

Ethical review and approval was not required for the study on human participants in accordance with the local legislation and institutional requirements. The patients/participants provided their written informed consent to participate in this study. Written informed consent was obtained from the individual(s) for the publication of any potentially identifiable images or data included in this article.

## Author contributions

MH and GU: writing – original draft and subsequent revisions. GU and GF: data collection, supervision, writing – review and editing, also the treating doctors. PP, MS, PA, SF: review and editing, supervision. All authors contributed to the article and approved the submitted version.

## Supplementary material

Supplementary Figure 1 Flowchart of the patient timeline. CT, computed tomography; MRI, magnetic resonance imaging; IGRT, image-guided radiotherapy; VMAT, volumetric modulated arc therapy.
