# Supplementary material for: Case Report: Adjuvant image-guided radiation therapy reduces surgical invasiveness in malignant peripheral nerve sheath tumors
Source: Front Oncol. 2023 Apr 21;13:1129537. doi: 10.3389/fonc.2023.1129537 (PMC10167840; doi:10.3389/fonc.2023.1129537)
Supplement: Supplementary Figure 1 — Flowchart of the patient timeline. CT, computed tomography; MRI, magnetic resonance imaging; IGRT, image-guided radiotherapy; VMAT, volumetric modulated arc therapy. [file DataSheet_1.pdf]

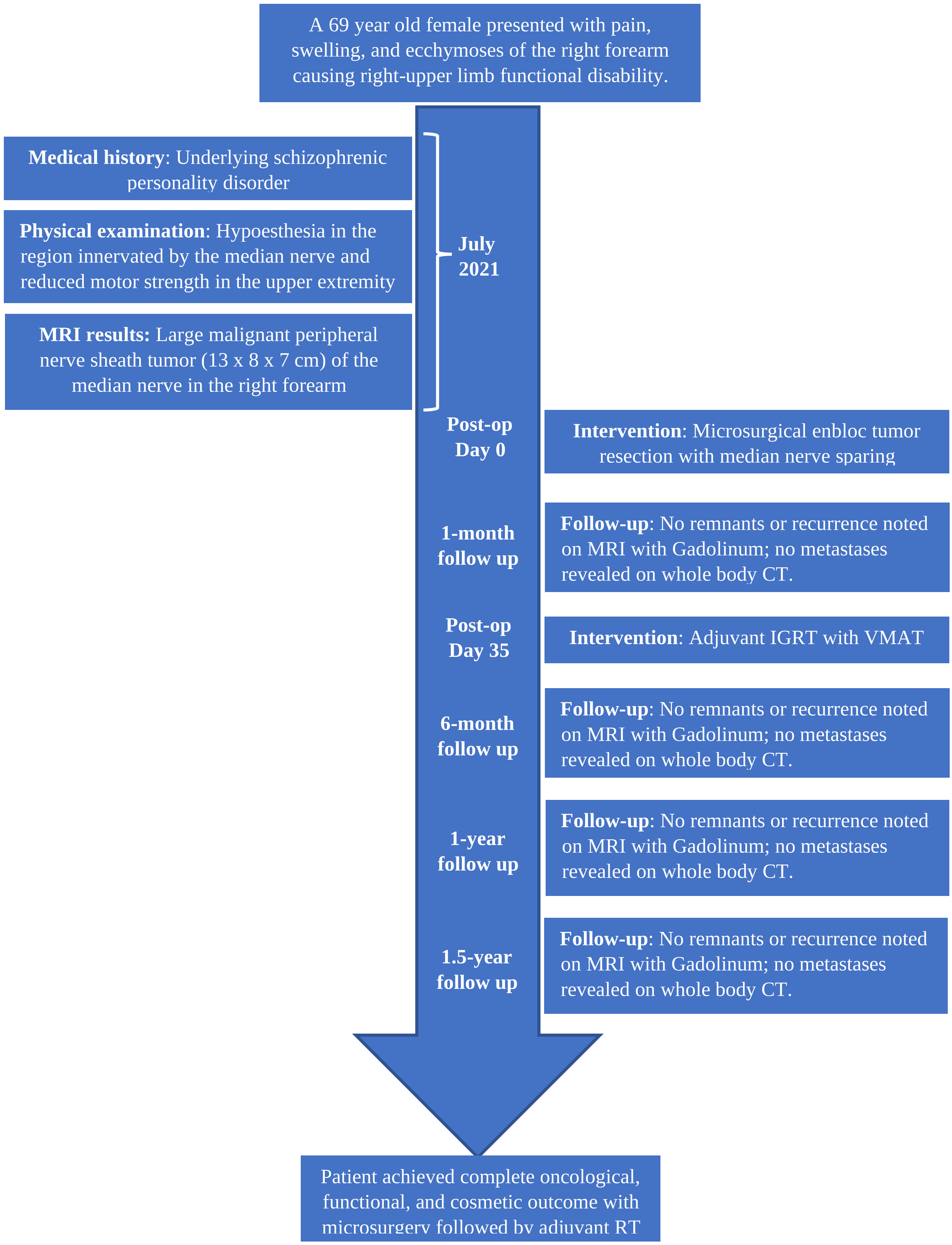
A 69 year old female presented with pain, swelling, and ecchymoses of the right forearm causing right-upper limb functional disability.
Medical history: Underlying schizophrenic personality disorder
Physical examination: Hypoesthesia in the region innervated by the median nerve and reduced motor strength in the upper extremity
MRI results: Large malignant peripheral nerve sheath tumor (13 x 8 x 7 cm) of the median nerve in the right forearm
July 2021
Post-op Day 0
Intervention: Microsurgical enbloc tumor resection with median nerve sparing
1-month follow up
Follow-up: No remnants or recurrence noted on MRI with Gadolinum; no metastases revealed on whole body CT.
Post-op Day 35
Intervention: Adjuvant IGRT with VMAT
6-month follow up
Follow-up: No remnants or recurrence noted on MRI with Gadolinum; no metastases revealed on whole body CT.
1-year follow up
Follow-up: No remnants or recurrence noted on MRI with Gadolinum; no metastases revealed on whole body CT.
1.5-year follow up
Follow-up: No remnants or recurrence noted on MRI with Gadolinum; no metastases revealed on whole body CT.
Patient achieved complete oncological, functional, and cosmetic outcome with microsurgery followed by adjuvant RT
